# Early implant‐associated osteomyelitis results in a peri‐implanted bacterial reservoir

**DOI:** 10.1111/apm.12597

**Published:** 2016-10-05

**Authors:** Louise Kruse Jensen, Janne Koch, Bent Aalbæk, Arshnee Moodley, Thomas Bjarnsholt, Kasper Nørskov Kragh, Andreas Petersen, Henrik Elvang Jensen

**Affiliations:** ^1^ Department of Veterinary Disease BiologyUniversity of CopenhagenFrederiksbergDenmark; ^2^ Department of Experimental MedicineUniversity of CopenhagenCopenhagenDenmark; ^3^ Costerton Biofilm CenterInstitute of Immunology and MicrobiologyUniversity of CopenhagenCopenhagenDenmark; ^4^ Department of Clinical MicrobiologyCopenhagen University HospitalCopenhagenDenmark; ^5^ Statens Serum InstitutCopenhagenDenmark

**Keywords:** Implant‐associated osteomyelitis, *S. aureus*, histopathology, animal experiment

## Abstract

Implant‐associated osteomyelitis (IAO) is a common complication in orthopedic surgery. The aim of this study was to elucidate how deep IAO can go into the peri‐implanted bone tissue within a week. The study was performed in a porcine model of IAO. A small steel implant and either 10^4^ CFU/kg body weight of *Staphylococcus aureus* or saline was inserted into the right tibial bone of 12 pigs. The animals were consecutively killed on day 2, 4 and 6 following implantation. Bone tissue around the implant was histologically evaluated. Identification of *S. aureus* was performed immunohistochemically on tissue section and with scanning electron microscopy and peptide nucleic acid *in situ* hybridization on implants. The distance of the peri‐implanted pathological bone area (PIBA), measured perpendicular to the implant, was significantly larger in infected animals compared to controls (p = 0.0014). The largest differences were seen after 4 and 6 days of inoculation, where PIBA measurements of up to 6 mm were observed. Positive *S. aureus* bacteria were identified on implants and from 25 μm to 6 mm into PIBA. This is important knowledge for optimizing outcomes of surgical debridement in osteomyelitis.

Implant‐associated osteomyelitis (IAO) is among the most severe orthopedic conditions 1. The absolute number of IAO cases is increasing, owing to the growing number of patients with bone implants 2. In the United States of America, the infection rate is 5–15% in fracture fixation devices and 0.3–5% in joint prosthesis 3, 4. Treatment of IAO can include surgical debridement, removal of implants and long‐lasting antimicrobial therapy, and calls for a multidisciplinary approach 1. Nevertheless, treatment failure is common. Despite removal of the infected device and extensive debridement, there is a high risk of re‐infection and prolonged use of postoperative antibiotics 5. In two different retrospective studies, treatment failure rates of IAO have been estimated to 41.8 and 58.2 percent, respectively 6, 7. An explanation for the infections and re‐infections of IAO has been suggested to be bacterial survival in the peri‐implanted bone tissue 8.

Insertion of orthopedic implants is an equipment requiring process that involves drilling and often the use of bone cements. A by‐product of these procedures is the generation of heat resulting in osteonecrosis 9. The necrotic osteocytes lose their inhibitory effects on osteoclasts leading to increased osteoclast activity and thereby bone resorption 10. Aside from resorption of dead bone, the process of osseointegration of an implant is especially dependent on the ingrowth of osteoblasts and mesenchymal stem cells 11. Along with osteonecrosis, bone resorption and osseointegration, a foreign body response also occurs around the implant 12. All these cellular changes make the peri‐implanted bone tissue a perfect *locus resistentiae minoris* for bacterial infection 13.

The aim of this study was to answer the following question: how deep is a bacterial infection going in the peri‐implanted bone tissue, in a case of IAO, within a week? The question was addressed in a porcine model of IAO in which evaluation of the entire infected bone is possible after a fixed period of time, compared to surgical biopsies from humans. Furthermore, it is highly relevant to use pigs for modeling of infectious diseases in humans, like IAO, as the porcine immune system shows a higher degree of similarity when compared to rodents and rabbits 14.

## Material and Methods

### Study design

The study is a descriptive study based on mainly microscopic observations in bone tissue and on orthopedic implants, obtained from a porcine model of IAO. The model is based on tibial insertion of a small steel implant combined with inoculation of *Staphylococcus aureus* bacteria or sterile saline. Twelve pigs of 30 kg body weight, obtained from a specific pathogen‐free (SPF) herd, were divided into three groups based on their time of killing (Table 1). The Danish Animal Experimental Inspectorate approved the protocol (license No. 2013/15‐2934‐00946).

**Table 1:** Summary of design and results in a porcine model of implant‐associated osteomyelitis

Animals			Summary of results				
Group	Inoculum CFU/Kg BW af *S. aureus* (*Spa*‐type)	Time of killing PI (Days)	Microbiology of swab from implant cavity (*Spa*‐type)	IHC detection of *S. aureus* in PIBA	Detection of *S. aureus* on implant surface by PNA FISH	Detection of biofilm on implant surface by SEM	Score of neutrophils in PIBA^13^
A	Saline	2	*S. aureus* (not *Spa*‐typed)	No	*–*	*–*	3+
	Saline	2	*S. aureus* (t034)	No	No	*–*	2+
	10^4^ (t1333)	2	*S. aureus* (t1333)	Yes	No	*–*	3+
	10^4^ (t1333)	2	*S. aureus* (t1333)	Yes	*–*	Yes	3+
B	Saline	4	*S. aureus* (not *Spa*‐typed)	No	*–*	*–*	2+
	Saline	4	*S. aureus* (t034)	Yes	No	*–*	3+
	10^4^ (t1333)	4	*S. aureus* (t1333)	Yes	No	*–*	3+
	10^4^ (t1333)	4	*S. aureus* (t1333)	Yes	*–*	Yes	3+
C	Saline	6	*S. aureus* (not *Spa*‐typed)	No	*–*	*–*	2+
	Saline	6	*S. aureus* (t1430)	No	*–*	Yes	2+
	10^4^ (t1333)	6	*S. aureus* (t1333)	Yes	*–*	Yes	3+
	10^4^ (t1333)	6	*S. aureus* (t1333)	Yes	Yes	*–*	2+

BW, body weight; PI, postinfection; IHC, immunohistochemistry; PIBA, peri‐implanted pathological bone area; PNA FISH, peptide nucleic acid *in situ* fluorescence hybridization; SEM, scanning electron microscopy.

### Experimental surgery and inoculum

Animals were anesthetized 15 and a tibial implant was inserted (K‐wire 2 × 20 mm) 1 cm below the growth plate of the right tibia. The procedure was recently described 16. The bacterial inoculum or saline was injected around the implant, before closure of the periost, subcutis and skin 16. The inoculating *S. aureus* strain was a pathogenic porcine strain, previously used in porcine models of osteomyelitis 17. The strain was prepared as described by Johansen et al. 18 and diluted with 0.9% sterile isotonic saline to obtain an inoculation dose of 10^4^ colony forming units (CFU)/kg BW in a final volume of 10 μL.

### Postoperative care of pigs

The pigs were daily monitored throughout the experiment by skilled personal. A body temperature above 41 °C, impaired ability to stand and anorexia were set as human endpoints. The pigs received intramuscular injections (0.1 mg/kg BW) of buprenorphine (Temgesic 0.3 mg/mL, Schering‐Plough, Heist‐op‐den‐Berg, Belgium) every 6–8 h. The pigs did not receive local or systemic antibiotic treatment, which is applied to human patients under therapy, as this would have hampered the focus of the study, that is, the ability to spread within the bone of the inflammation and infection.

### Pathology

Following killing after 2, 4 and 6 days, the implant was removed from the implant cavity using a sterile lancet and collected for scanning electron microscopy (SEM) and peptide nucleic acid fluorescence *in situ* hybridization (PNA FISH). From all pigs, the right tibia was dissected free and decalcified. However, in group C animals, the right tibial bone was sagittal sectioned through the implant cavity before decalcification. Following decalcification, the proximal end of right tibial bone, containing the implant cavity, was cut into five sagittal pieces 3–4 mm each. Afterward, the bone pieces were processed routinely and embedded in paraffin wax. Sections (4–5 μm) were stained with hematoxylin and eosin (HE) and special stained with phosphotungstic acid hematoxylin (PATH) and Masson's trichrome for demonstration of fibrin and collagen, respectively. Bone tissue with pathological changes, around the implant cavity, was defined as the peri‐implanted pathological bone area (PIBA). The largest size of PIBA was measured perpendicularly to the implant cavity on the most representative section. On the same section, the scoring system developed by Pandey el al. 19 was used to define whether an infection occurred. Pandey found that the presence of 2+ or more (more than one neutrophil granulocyte per high power field (400) on average after examination of at least 10 high power fields) in periprosthetic tissue, distinguishing between septic and aseptic loosening of prosthesis and bone implants in humans. The scores were as follows: 0 = absent; 1+ less than 1 cell on average per high power filed; 2+ = 1–5 cells on average per high power fields; 3+ = >5 cells on average per high power fields.

### Microbiology

Cotton swabs were taken from the implant cavity after removal of the implant. Swabs were processed and characterized as previously described 20, and selected bacterial isolates were *Spa*‐typed 21.

### Immunohistochemistry of bone tissue

Tissue sections of 4 μm were prepared and processed for indirect *in situ* identification of *S. aureus* with immunohistochemistry. Primary *S. aureus‐*specific antibodies (ab37644; Abcam, Cambridge, UK, diluted 1:1000 in 5% swine serum) were used 18. The size of the largest observed *S. aureus‐*positive aggregates and their distance to the implant cavity were estimated on each section, by measuring the length directly on the immunohistochemistry (IHC) sections.

### Scanning electron microscopy and peptide nucleic acid fluorescence *in situ* hybridization of implants

Selected implants were examined with scanning electron microscopy (SEM) and peptide nucleic acid fluorescence *in situ* hybridization (PNA FISH) (Table 1). The implants were placed in 2, 5% glutaraldehyde or formalin for SEM and PNA FISH, respectively. The samples for SEM were rinsed three times in 0.15 M sodium phosphate buffer (pH 7.4); specimens were postfixed in 1% OsO4 in 0.12 M sodium cacodylate buffer (pH 7.4) for 2 h. Following a rinse in distilled water, the specimens were dehydrated to 100% ethanol and critical point dried (Balzers CPD 030 instrument, Leica Microsystems, Wetzlar, Germany) using CO_2_. The specimens were subsequently mounted on stubs, using colloidal coal as an adhesive, and sputter coated with gold (Polaron SEM E5000 coating unit). Specimens were examined with a Philips FEG30 scanning electron microscope operated at an accelerating voltage of 2 kV. The samples for PNA FISH were carefully rinsed in sterile saline for 5 min. Samples were placed in a dish (depth 1 mm, diameter 10 mm) and covered by 100 μLn PNA FISH‐specific *S. aureus* probe (Advandx, Woburn, Massachusetts, USA). Samples were incubated at 55 °C for 90 min. Subsequently, dishes were submersed for 30 min in 55 °C warm wash buffer (4 mL 60× wash buffer (Advandx) to 240 mL miliQ water). Afterward, the samples were air‐dried in the dark followed by covering by 3 mM DAPI solution (Life Technologies, Carlsbad, California, USA) for 15 min at room temperature. Excess DAPI was removed by gently rinsing with PBS (the Substrate Department at the Panum Institute, Denmark). All observations were performed using a Zeiss LSM 710 confocal laser scanning microscope (Oberkochen, Germany).

### Statistics

An unpaired t‐test was used to analyze PIBA measurements between control and infected animals (GraphPad Software inc, version 7, LaJolla, California, USA).

## Results

### Clinical observations

All animals were healthy when entering the study, but became lame on the operated leg after surgery and up until killing. The degree of lameness was low, as the animals were able to use the leg and walk around freely. One of the infected Group B animals had intermittent elevated body temperature. All animals ate and drank normally before and during the experiment.

### Macroscopic pathology

**Figure 1:**
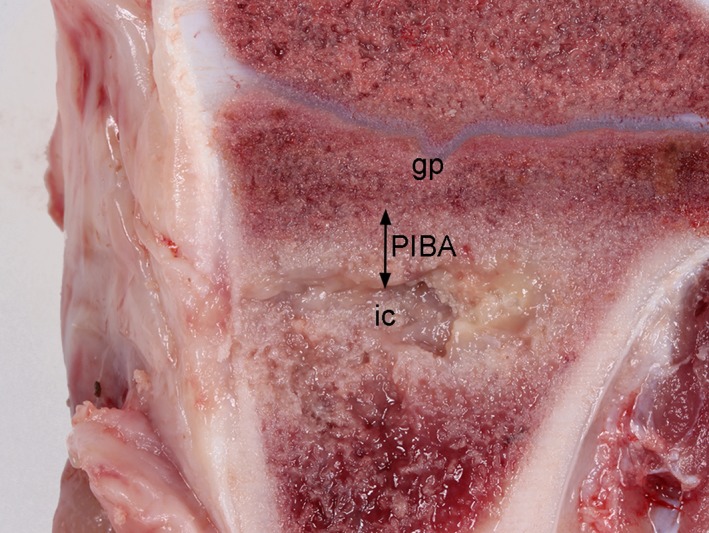
Macroscopic pathological changes in a porcine tibial bone 6 days after *S. aureus* inoculation and insertion of a steel implant. The implant was inserted just below the grow plate (gp) in the trabecular bone tissue. The implant has been removed on the picture, presenting the implant cavity (ic). Purulent exudation is seen in the implant cavity and within the surrounding bone tissue. The peri‐implanted bone tissue with pathological changes is referred to as the peri‐implanted pathological bone area (PIBA).

Thick purulent material was seen within the implant cavity in the infected animals following 4 and 6 days. In all control pigs, and pigs infected for 2 days, serohemorrhagic fluid was present. Following sagittal section of the inoculated tibial bone from the two infected Group C animals, signs of osteomyelitis were seen around the implants as purulent and sequestered trabecular bone tissue (Fig. 1).

### Histopathology

**Figure 2:**
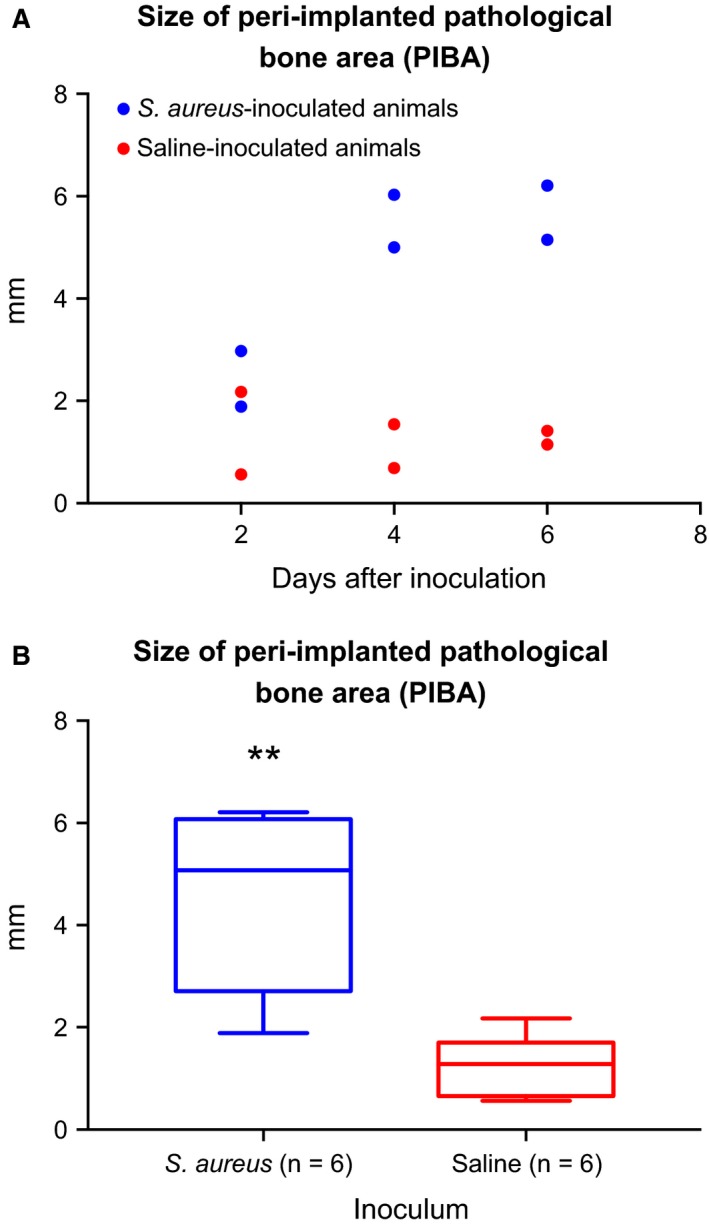
(A) The size in mm of the peri‐implanted pathological bone area (PIBA) in a porcine model of implant‐associated osteomyelitis 2, 4 and 6 days after tibial insertion of a steel implant and injection of *Staphylococcus aureus* (blue) or saline (red). PIBA was measured perpendicular from the implant cavity and until normal tissue architecture occurred. (B) Grouping of animals inoculated with *Staphylococcus aureus* and animals inoculated with saline. The figure shows the size in mm of the peri‐implanted pathological bone area (PIBA). Boxes, 95% confidence interval; whiskers, min. and max. value: bold line, mean value. The size of PIBA was significantly (** = P ≤ 0.001) increased in pigs inoculated with *S. aureus*.

The neutrophil granulocyte score counted inside PIBA and the size of PIBA is presented in Table 1 and Fig. 2A, respectively. The size of PIBA showed no marked difference between infected and control animals after 2 days from surgery with a mean difference of 0.7 mm (Fig. 2A). However, in Groups B and C, the mean differences between infected and control animals were 4.3 mm and 4.4 mm, respectively (Fig. 2A). The p‐value of the difference in PIBA size between control (n = 6) and infected animals (n = 6) was 0.0014 (Fig. 2B). The largest registered PIBA value for day 4 (Group B) and day 6 (Group C) was 6.0 mm and 6.21 mm, respectively (Fig. 2A).

**Figure 3:**
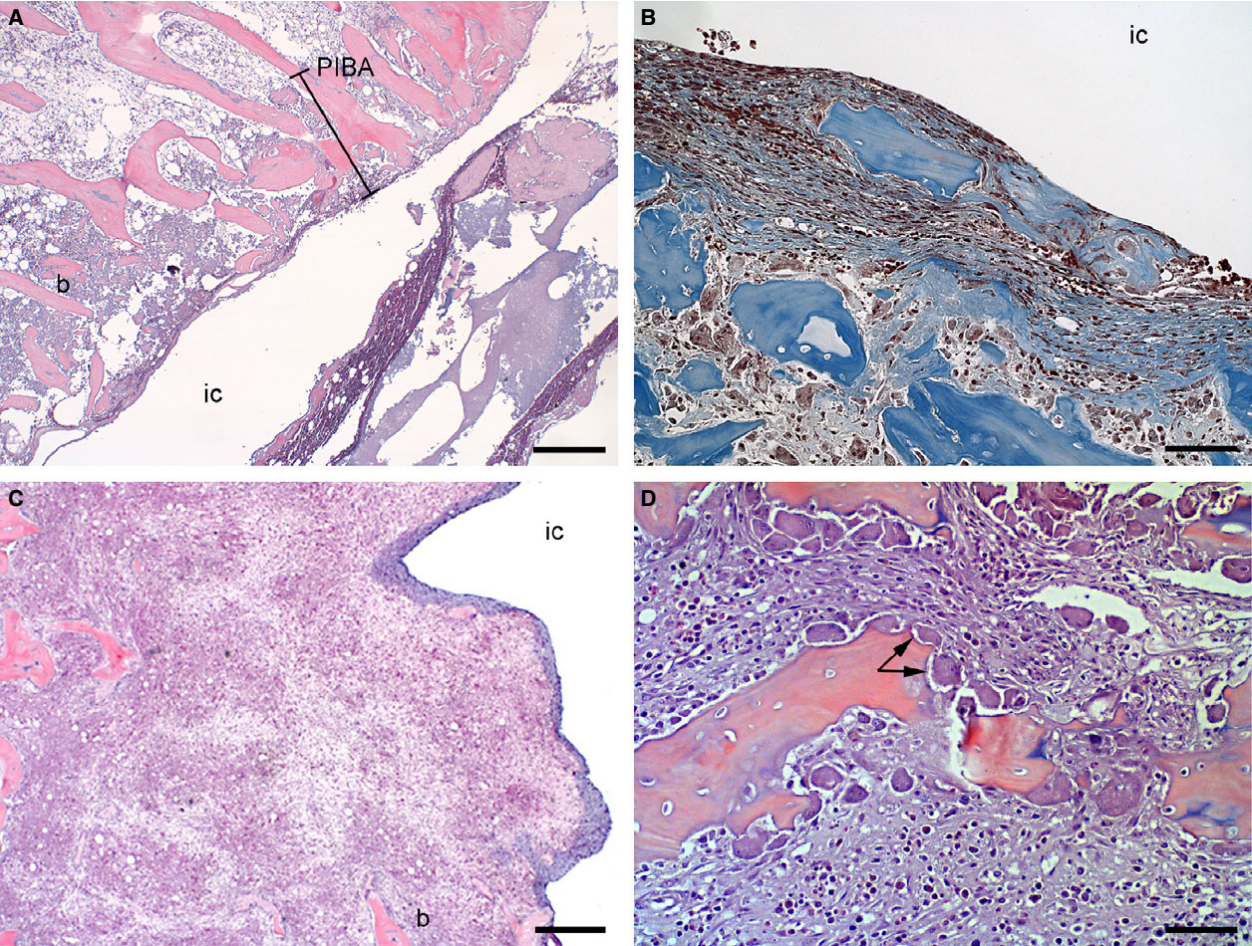
Overview of histopathological bone changes in porcine tibial bones following 2 (A) and 6 (B, C and D) days after *S. aureus* or saline inoculation and insertion of a steel implant. Pictures A and B represent pigs inoculated with saline, and pictures C and D represent pigs inoculated with *S. aureus*. IC: implant cavity. Picture A: an overview of the peri‐implanted pathological bone area (PIBA) after 2 days of inoculation. HE, bar = 400. Picture B: a thin area of primarily fibroblasts and collagen was seen on the surface toward the implant cavity. Masson's trichrome, bar = 250. Picture C: a large area of fibroblasts, collagen, giant cells and macrophages was seen toward the implant cavity. Almost no bone (b) tissue was left within PIBA. HE, bar = 300. Picture D: a dominant finding between infected and control pigs after day 4 of inoculation was a substantial presence of active osteoclasts (arrow). HE, bar = 200.

The following description of pathomorphological changes is correlated with the trabecular tissue only. In Group A, PIBA was a mix of erythrocytes, bone marrow leukocytes, necrotic trabecular tissue (with empty lacuna), neutrophils and fibrin exudation (Fig. 3A). However, more neutrophils and extensive fibrin exudation were seen in the infected animals. At 4 and 6 days after inoculation (Groups B and C), a cellular layer of elongated fibroblasts, neutrophils, macrophages and giant cells were seen toward the implant cavity (Fig. 3B). This layer was surrounded by osteonecrotic trabecular bone intermingled with the same cell types. The most pronounced pathomorphological findings within PIBA of infected Groups B and C animals were massive extension of the cellular layer and active osteoclasts seen in resorptions lacuna of the necrotic bone trabecular (Fig. 3C,D). In the periphery of PIBA, fibroblasts and associated new collagen could be seen, although more pronounced in infected animals compared to controls. Generally, almost no bone tissue was present within PIBA of infected Group C animals.

### Localization of bacteria in bone tissue

**Figure 4:**
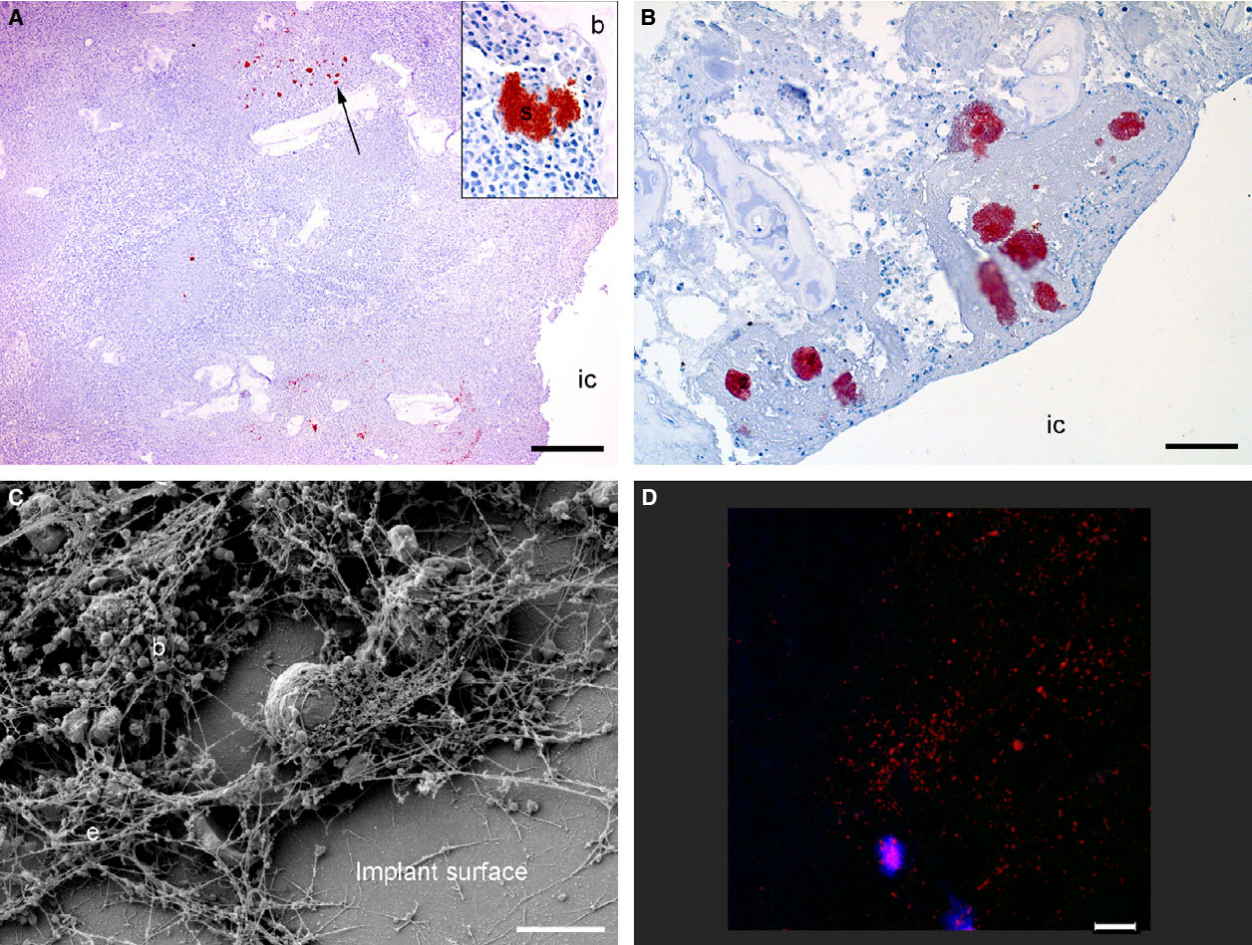
Visualization of bacteria inside bone tissue and on the surface of steel implants from a porcine model of implant‐associated osteomyelitis inoculated with *S. aureus* or saline. IC: implant cavity. Picture A: six days after bacterial inoculation, *S. aureus‐*positive bacteria (arrow) were seen in the peri‐implanted pathological bone area (PIBA). Insert; Close‐up of a positive *S. aurous* colony, IHC staining for *S. aureus*, bar = 300. Picture B: four days after inoculation with saline, *S. aureus‐*positive bacteria were seen enclosed just inside PIBA. These bacteria are supposed to be a result of self‐contamination, as the *Spa* type of the implant cavity revealed another *S. aureus* strain as the one used for inoculation, IHC staining for *S. aureus*, bar = 200. Picture C: scanning electron microscopy (SEM) of an implant surface following 4 days of inoculation with *S. aureus* showing bacteria (b) and extracellular material (e), bar = 5 μm. Picture D: peptide nucleic acid fluorescence *in situ* hybridization (PNA FISH) of implant surface following 6 days of inoculation with *S. aureus*. Bacteria positive for the *S. aureus* probe light up in red and leukocytes in blue, bar = 20.

Bacteria were not identified by IHC in cortical bone tissue of any animal. Immunopositive *S. aureus* bacteria were present in all infected pigs of Groups A, B and C within both the exudate of the implant cavity and within PIBA (Table 1 and Fig. 4A). Additionally, *S. aureus* bacteria were also detected in one control animal of Group B within BIPA (Table 1 and Fig. 4B). IHC‐positive *S. aureus* bacteria were not identified in the control animals of Groups A and C. On the tissue section representing the center of PIBA, the size of the largest *S. aureus‐*positive aggregate stayed between 25 and 84 μm and was located from 25 to 2000 μm within PIBA regardless of the time of inoculation. Moreover, bacteria were found on two consecutively sections from distant bone pieces (2–3 mm each), not covering the implant cavity, in one infected animal of Groups A and B, each. All bacterial aggregates inside PIBA were seen within the cellular zone or adjacent to osteonecrosis.

### Microbiology

All swab and *Spa*‐typing results are presented in Table 1.

### Implants SEM and PNA FISH

By SEM, coccoid bacteria were observed attached to the surface of all implants from both control and infected animals in the form of biofilm. The areas of biofilm were defined to isolated islands on the implant surface. In general, the bacteria were found embedded in a biomass of extracellular matrix, leukocytes and erythrocytes (Fig. 4C). Attached leukocytes appeared to be more dominant on implants of infected animals. By PNA FISH, *S. aureus* could be identified on one implant (Table 1 and Fig. 4D).

## Discussion

This study shows that pathomorphological changes and infecting bacteria of IAO can go up to 6 mm into the surrounding bone tissue within a week, in a porcine model (Fig. 2). The surrounding bone tissue was described as PIBA. Within PIBA, *S. aureus* bacteria could be identified both adjacent to and distant from the implant. However, despite bacteria in PIBA (shown with IHC), in the exudate within the implant cavity (shown with microbiology of swabs) and within the biofilm on the implants (shown with PNA FISH and SEM), bacterial aggregates could not be demonstrated laying on the surface of PIBA, that is, facing the implant cavity.

The present observations of peri‐implanted bacteria are supported by a study of subcutaneous implant‐associated infections (IAI) in a mouse model, showing that bacteria (*Staphylococcus epidermidis*) could be found within the tissue at a certain distance from the implant 22, 23. In orthopedic infections, bacteria are usually discussed in the context of biofilm formation directly on the implant 24. However, case reports of IAO have also confirmed that peri‐implanted bone tissue may contain pathogenic bacteria 25, 26. Recently, by the same method as in this study 27, the size of bacterial colonies within peri‐implanted bone tissue from patients with osteomyelitis has been estimated to range between 5 and 50 μm. In general, the maximal size of an *in vivo* biofilm has been estimated to be 200 μm 27. All measurements of *S. aureus* colonize within peri‐implanted bone tissue of the present porcine model were below 84 μm. Therefore, the size of the observed bacterial colonies can be accepted as discriminative to human cases.

The Infectious Disease Society of America (IDSA) has recently proposed five criteria for the diagnosis of periprosthetic infections 28. The infected pigs from this study fulfill criteria 2 (pus around the implant), 3 (histopathological evidence of inflammation) and 5 (positive intraoperative cultures). Additionally, the control animals also fulfilled criteria 3 and 5. However, *Spa*‐typing results indicated that the control animals contaminated and infected themselves. The *Spa* types observed among control pigs isolates are typically seen in commensal porcine *S. aureus* strains 29 and differed from the *Spa* type used for inoculation 17. Self‐contamination of the control animals thus highlights the hypothesis of the peri‐implanted area as a *locus resistentiae minoris*; that is, a minimal number of bacteria can colonize an implant and the surrounding tissue. As *S. aureus* is the most common pathogen associated with IAO, and an independent risk factor of treatment failure 6, this bacterium was used. The selected porcine *S. aureus* strain was chosen, due to its ability to induce bone infection and inflammation 17, 18. The dose of 10^4^ CFU/kg BW was estimated to be among the lowest infective dose, based on a former dose–response study using the same porcine *S. aureus* strain 18.

All animals scored 2+ or 3+ in number of neutrophils. The scoring system for the number of neutrophils was originally used on tissue section from cases of chronic low‐grade IAO in humans 19. Specific neutrophil number cutoffs for high‐grade IAO, to which infected animals of this study may belong, has not been established 30.

Although the number of infected and control animals killed on each time point is limited, grouping of pigs as either being infected (n = 6) or control (n = 6) animals allowed a statistical comparison of PIBA size (Fig. 2B). As significance was observed between these two groups, the group size was acceptable, that is, including more pigs could be a waste of animals. It was not possible to do statistical calculations between infected and control animals of each time points. However, the values from each time points can give an estimate of the variance and effect size in power/sample size calculations. This might be relevant for further experiments aiming to study effects of surgical or medical treatments in the porcine model. The histopathological examinations of all animals clearly demonstrated trabecular osteonecrosis around the implant cavity due to drilling of the bone prior to insertion of the implant. Additionally, the injection of *S. aureus* bacteria resulted in noticeable pathomorphological changes between infected and control animals, visualized by the size of PIBA (Fig. 2A). The most obvious changes within infected animals were bacterial aggregates and accumulations of inflammatory cells, giant cells, active osteoclasts and fibroblasts. Thus, the present injection of bacteria, during insertion of the bone implants, resulted in biofilm formation on the implants and a peri‐implanted bacterial reservoir directing bone pathomorphology. A peri‐implanted bacterial reservoir might hamper a fast diagnosis and therapeutic debridement in cases of IAO. Furthermore, when located in the peri‐implanted bone tissue, *S. aureus* bacteria can form biofilm 24, small colony variants 31 and be internalized by osteoblasts 31, three situations which favor persistence of the bacteria and infection.

The present porcine model of IAO showed that bacterial aggregation occurred on the surface of implants and within the surrounding bone tissue within 6 days of infection. Thus, it is important to notice that peri‐implanted bone tissue shortly after implantation can serve as a reservoir for infecting bacteria. This is important knowledge for optimizing outcomes of surgical debridement in IAO. In clinical situations of acute IAO, that is, lasting for less than 3–4 weeks, simple lavage has been recommended 28. However, as this study shows, this approach might be problematic if the bacteria already within 1 week can be located half a centimeter into the surrounding bone tissue.

We thank Betina Andersen and Elizabeth Petersen for their excellent laboratory assistance and the Core Facility for Integrated Microscopy at Copenhagen University for assistance with SEM. This study was financed by grant no. 4005‐00035B from the Danish Medical Research Council. No conflict of interests was declared.
